# Potential of promotion of alleles by genome editing to improve quantitative traits in livestock breeding programs

**DOI:** 10.1186/s12711-015-0135-3

**Published:** 2015-07-02

**Authors:** Janez Jenko, Gregor Gorjanc, Matthew A Cleveland, Rajeev K Varshney, C. Bruce A Whitelaw, John A Woolliams, John M Hickey

**Affiliations:** The Roslin Institute and Royal (Dick) School of Veterinary Studies, The University of Edinburgh, Easter Bush, Midlothian, Scotland UK; Genus plc.,100 Bluegrass Commons Blvd., Suite 2200, Hendersonville, TN 37075 USA; International Crop Research Institute for the Semi-Arid Tropics (ICRISAT), Patancheru, India

## Abstract

**Background:**

Genome editing (GE) is a method that enables specific nucleotides in the genome of an individual to be changed. To date, use of GE in livestock has focussed on simple traits that are controlled by a few quantitative trait nucleotides (QTN) with large effects. The aim of this study was to evaluate the potential of GE to improve quantitative traits that are controlled by many QTN, referred to here as promotion of alleles by genome editing (PAGE).

**Methods:**

Multiple scenarios were simulated to test alternative PAGE strategies for a quantitative trait. They differed in (i) the number of edits per sire (0 to 100), (ii) the number of edits per generation (0 to 500), and (iii) the extent of use of PAGE (i.e. editing all sires or only a proportion of them). The base line scenario involved selecting individuals on true breeding values (i.e., genomic selection only (GS only)-genomic selection with perfect accuracy) for several generations. Alternative scenarios complemented this base line scenario with PAGE (GS + PAGE). The effect of different PAGE strategies was quantified by comparing response to selection, changes in allele frequencies, the number of distinct QTN edited, the sum of absolute effects of the edited QTN per generation, and inbreeding.

**Results:**

Response to selection after 20 generations was between 1.08 and 4.12 times higher with GS + PAGE than with GS only. Increases in response to selection were larger with more edits per sire and more sires edited. When the total resources for PAGE were limited, editing a few sires for many QTN resulted in greater response to selection and inbreeding compared to editing many sires for a few QTN. Between the scenarios GS only and GS + PAGE, there was little difference in the average change in QTN allele frequencies, but there was a major difference for the QTN with the largest effects. The sum of the effects of the edited QTN decreased across generations.

**Conclusions:**

This study showed that PAGE has great potential for application in livestock breeding programs, but inbreeding needs to be managed.

**Electronic supplementary material:**

The online version of this article (doi:10.1186/s12711-015-0135-3) contains supplementary material, which is available to authorized users.

## Background

Livestock breeding programs aim at improving populations by increasing the genetic merit of traits of socioeconomic importance. These are typically quantitative traits that are affected by many quantitative trait nucleotides (QTN), most of which only have small effects [1]. Recent use of genome-wide markers has led to the implementation of genomic selection (GS) [2, 3], which attempts to capture the effect of QTN through associations between phenotypes and markers. GS is now routinely used in advanced animal breeding programs to drive genetic progress for quantitative traits [4–7]. The commercial success of GS will result in large datasets of phenotyped and densely genotyped individuals for several species. During the next decade, it is likely that these datasets will comprise many hundreds of thousands or millions of individuals with sequence level information [8]. Analysis of such datasets will enable the identification of large proportions of the QTN for quantitative traits. Increasing the frequency of favourable alleles at these QTN will be slow with conventional selection methods because quantitative traits are defined by many QTN and the low levels of recombination during meiosis limit the rate at which favourable alleles can occur together in selected individuals. Alternatives to these conventional selection methods will be required to overcome these limitations. An example of such a method, proposed in this work, is promotion of alleles by genome editing (PAGE); which has the potential to enable rapid increases in the frequency of favourable alleles. PAGE offers the opportunity to move genetic variation between individuals in a population much more freely since it enables individual QTN alleles to be moved independently of all other QTN alleles.

Genome editing (GE) is a technique for adding, deleting, or replacing a series of nucleotides in the genome of a cell. When these changes in the genome are made to the germline, they are permanent and are therefore transferred to future generations [9–11]. In recent decades, major advances have been made in the development of GE methods. The first successful editing of a mammalian genome was performed on mice in the 1980s [12], but the approach used was untargeted and thus the location of the modification could not be controlled. Later approaches that could target specific locations were developed, based on the use of homologous recombination [13]. These techniques also had low success rates because of the low occurrence of recombinations. This was overcome by techniques using specific nucleases, *i.e.*, molecular scissors, which cut DNA at specific, predetermined places. The more commonly known nucleases are zinc finger nuclease (ZFN), transcription activator-like effector nucleases (TALEN), and the clustered regulatory interspersed short palindromic repeats (CRISPR) associated system [14, 15]. These methods differ in the way they recognise a target location. ZFN and TALEN use DNA-protein binding sites, while CRISPR methods use RNA. ZFN-and TALEN-based methods are complex to use and costly, and therefore have limited usefulness in practice. In comparison, CRISPR-based methods are simpler to use and have become increasingly popular [16–19].

Currently, although these methods are extremely accurate (to the single target base pair), they lack efficiency. For example, in a recent study, ZFN and TALEN were used to edit a single locus in 500 pig embryos; of these, 55 piglets were born alive and among these, only five were homozygous and four were heterozygous for the edited allele, which resulted in a success rate of 16 % per piglet born [20]. In another study, the success rate was 68.8 % using CRISPR [21]. These studies indicate that the success rate of GE is improving, but there are still some unknown factors, especially with regard to unintended edits that occur at QTN that are not the target [22] and with regard to regulatory approval by governmental agencies for the use of GE in commercial livestock breeding. In spite of these uncertainties, it is possible that, in the near future, GE will have attained government approval and will be highly accurate and efficient. This would enable large numbers of edits to be performed for large numbers of individuals and therefore, it is appropriate for animal breeders who seek to improve quantitative traits to begin to consider how GE technology could be used to advance breeding programs.

In order to use GE to improve traits, the variants controlling the traits must be identified. QTN with large effects are easier to identify than QTN with small effects. Thus, to date, most applications of GE have focussed on large effect variants controlling qualitative as opposed to quantitative traits. For example, GE has been used to produce bovine fibroblasts edited for the polled allele [23], to generate pigs with a single base deletion in a gene that may confer resilience to African Swine Fever Virus [24], and to introduce mutations in the *myostatin* gene in sheep and cattle [25]. The suitability of GE as a tool for improving quantitative traits in livestock breeding programs, referred to herein as PAGE, is unknown because quantitative traits are influenced by many QTN, most of which have a small effect. One hypothesis is that quantitative traits may require large numbers of QTN to be edited before a major benefit is observed. Editing large numbers of QTN has yet to be achieved, but is a realistic proposition within a five to ten year timeframe. Also, large datasets of phenotyped and sequenced individuals would be required to identify large numbers of QTN for quantitative traits. An alternative hypothesis is that by focussing PAGE resources on small numbers of QTN, which have moderate effects and exist for quantitative traits, a relatively small number of edits could be used to increase the rate of genetic improvement for quantitative traits. Using a large dataset with 253 000 individuals, around 697 GWAS (genome-wide association studies) hits with moderate effects were discovered for human height, which cumulatively explained about 20 % of the heritability for this highly polygenic quantitative trait [26]. Datasets of this size, and even greater, are now becoming available in livestock breeding programs [8].

The objective of this study was to quantify the potential of GS supplemented by PAGE compared to genomic selection only (GS only) to increase the response to selection for quantitative traits in livestock breeding programs. Simulation of different strategies was used to quantify the potential of PAGE for a trait defined by 10 000 QTN. The results showed that compared to GS only, GS supplemented by PAGE enabled a much greater response to selection but that some strategies for PAGE resulted in much greater rates of inbreeding than GS only. The increase in response to selection was driven by the ability of GS supplemented by PAGE to increase the frequencies of favourable alleles at the QTN with larger effects faster than GS only.

## Methods

**Fig. 1:**
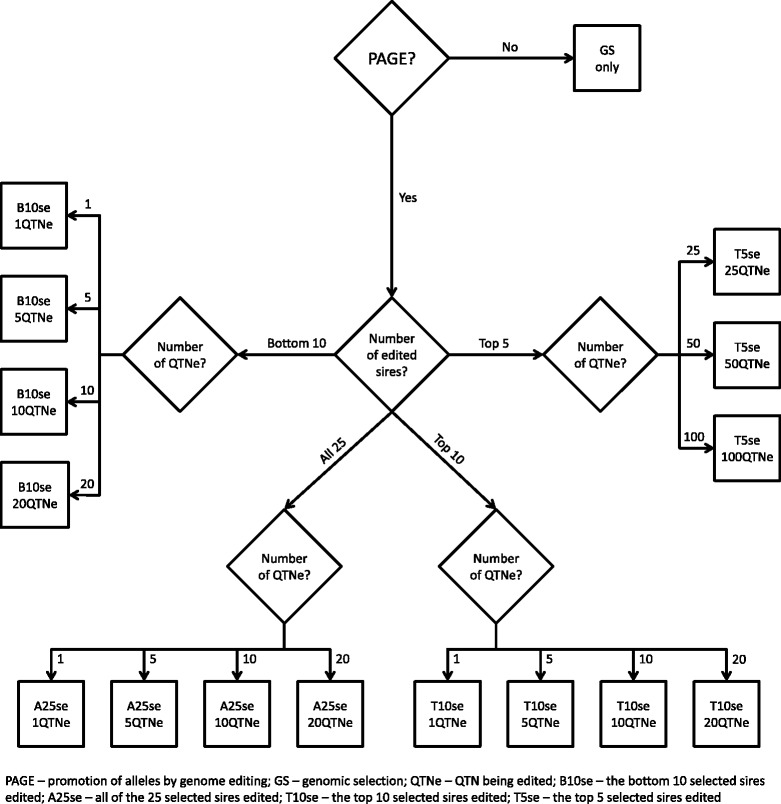
Overall design of the simulation

Simulations were used to evaluate the potential of PAGE to increase the response to selection for quantitative traits in livestock breeding programs. Ten replicates of several scenarios were performed with the overall simulation scheme in Fig. 1. The simulations were designed to: (i) generate whole-genome sequence data, (ii) generate QTN that affect phenotypes, (iii) generate pedigree structures for a livestock population, and (iv) test different selection and PAGE strategies. Conceptually, the simulation scheme was divided into a historical and a future component. The historical component represented historical evolution and recent historical breeding efforts up to the present day under the assumption that livestock populations have been evolving for tens of thousands of years, followed by 21 recent generations of modern animal breeding with selection on breeding values for the simulated trait only. The future component represented 20 future generations of modern animal breeding in which the breeders had different options and technologies at their disposal (i.e., PAGE and GS). The historical animal breeding generations were denoted generations–20 to 0 and the future animal breeding generations were denoted 1 to 20.

### Genome sequence simulation

Sequence data were generated using the Markovian Coalescent Simulator (MaCS) [27] and AlphaDrop [28] for 1000 haplotypes for each of ten chromosomes. The chromosomes, each 100 cM long and comprising 10^8^ base pairs, were simulated using a per site mutation rate of 2.5 × 10^−8^, and an effective population size (N_e_) that changed over time. Based on estimates for the Holstein cattle population [29], the Ne was set to 100 in the final generation of simulation and to 1256, 4350, and 43 500 at 1000, 10 000, and 100 000 generations ago, with linear changes in between. The resulting sequences had approximately 550 000 segregating sites in total.

### Simulation of QTN and phenotypes

We simulated a quantitative trait that had 10 000 QTN that were randomly sampled from the segregating sites, with the restriction that an equal number of QTN was sampled for each chromosome. These QTN had their allele effects sampled from a normal distribution with a mean of 0 and standard deviation of 1.0 divided by the square root of the number of QTN. The effects of QTN were in turn used to compute true breeding values (TBV) for the quantitative traits.

### Pedigree simulation

After the sequence and QTN simulation, a pedigree of 41 generations was simulated. In the first generation of the recent historical animal breeding population (denoted as generation–20), individuals had their chromosomes sampled from the 1000 simulated haplotypes. In later generations, individuals had their chromosomes sampled from parental chromosomes with recombination. Crossovers were simulated without interference. Each generation comprised 1000 individuals (500 males and 500 females), from which 25 males and 500 females were selected as parents of the next generation. Selection was based on TBV, because PAGE requires that at least some of the QTN were assumed known. When all QTN are known, the accuracy of GS is perfect. In this study, only 1000 individuals were generated in each generation and a dataset of this size would be insufficient to ensure accurate fine mapping of QTN or accurate estimation of breeding values in GS.

### Promotion of alleles by genome editing

**Fig. 2:**
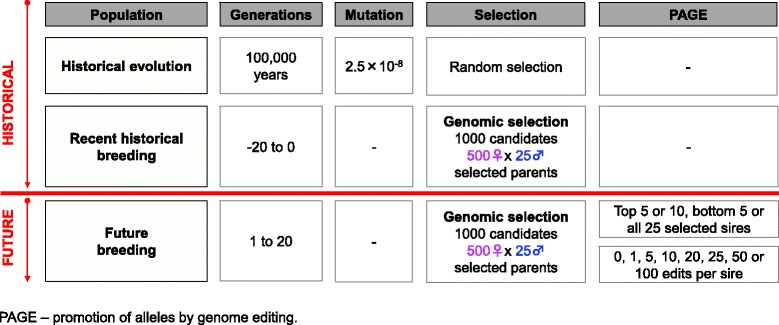
Simulated scenarios for promotion of alleles by genome editing

PAGE was not applied in the historical animal breeding generations (generations–20 to 0), but it was applied in the future animal breeding generations (1 to 20) for all except one of the scenarios, (Fig. 2). To apply PAGE, 25 sires were first selected on the basis of their TBV and then a subset of these sires was edited for 0, 1, 5, 10, 20, 25, 50, or 100 QTN. For each sire, the QTN with the largest allele substitution effect for which the sire was not already homozygous for the favourable allele were edited (edited QTN are denoted QTNe). It was assumed that QTN effects were known and only those QTN that segregated in generation 0 were considered to be QTN that could be edited to ensure that the same genetic variation was available in scenarios with and without PAGE. If QTN that segregated in generation–20 had been considered, the favorable alleles that were lost during the historical animal breeding generations would be available for scenarios with PAGE but not for scenarios without PAGE, which would give PAGE scenarios an unfair advantage.

The subsets of edited sires were: (i) all 25 sires (A25se); (ii) the 10 sires with the highest TBV (T10se); (iii) the 10 sires with the lowest TBV (B10se); and (iv) the five sires with the highest TBV (T5se). Scenario A25se was designed to generally assess the suitability of PAGE for increasing response to selection in quantitative traits. Scenarios T10se, B10se, and T5se were designed to determine whether differences in short-and long-term response to selection could be observed, as well as the impact of alternatives to distribute a limited amount of PAGE resources across the population. In total, these scenarios comprised 16 different sub-scenarios (Fig. 2). In the remainder of this paper, scenarios with PAGE are referred to as GS + PAGE and scenarios without PAGE are referred to as GS only.

### Quantification of the impact of PAGE

The potential of PAGE to increase response to selection for quantitative traits in livestock breeding programs was evaluated by the cumulative response to selection at each generation. Cumulative response to selection was calculated as:

$$ \raisebox{1ex}{$\left(\overset{-}{TB{V}_{curr}}-\overset{-}{TB{V}_{base}}\right)$}\!\left/ \!\raisebox{-1ex}{${\sigma_{TBV}}_{base}$}\right., $$

Where $\overline{TB{V}_{curr}}$ is the mean TBV of the current generation and $\overline{TB{V}_{base}}$ and ${\sigma_{TBV}}_{{}_{base}}$ are the mean and standard deviation of TBV of base generation–20, respectively. The unit of cumulative response to selection was standard deviation of TBV in the base generation. Generation–20 was used as the base generation in order to observe the genetic improvement since the start of recent historical breeding, in which GS only was used, and to compare it to future breeding, in which GS only or GS + PAGE was used. Cumulative response during future breeding was also evaluated with $\overline{TB{V}_{base}}$ set equal to the mean of TBV in generation 0 in order to evaluate differences between GS only and GS + PAGE since the start of future breeding activities. Unless otherwise stated, the results described below refer to situations in which generation 0 was the base generation. To investigate the consequences of using PAGE, changes in allele frequencies were monitored for all QTN that were segregating in generation 0 and for the 20 QTN with the largest effects that were segregating in generation 0. Changes in genic variance due to the changes in allele frequency at these QTN was also calculated at each generation. The genic variance [30] was calculated as:

$$ {\displaystyle {\sum}_i^n2{p}_i\left(1-{p}_i\right){\alpha}_i^2}, $$

where *p*_*i*_ is the frequency of the favourable allele at the *i*-th QTN and *α*_*i*_ is the allele substitution effect. The genic variance was calculated for all QTN and for the 20 QTN with the largest effects. The number of distinct QTNe in each generation and the effect sizes of all QTN that we edited in each generation were also recorded. A QTN was considered edited if it was edited for at least one sire. Finally, the average pedigree-based inbreeding coefficient was calculated for each generation.

## Results

### Response to selection

**Fig. 3:**
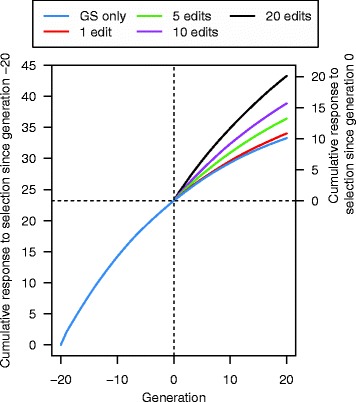
Cumulative response to selection across 21 generations of recent historical breeding based on genomic selection only (GS only) and 20 generations of future breeding based on GS only or genomic selection plus the promotion of alleles by genome editing (GS + PAGE) when different numbers of QTN (1, 5, 10, or 20) were edited for all 25 sires

**Fig. 4:**
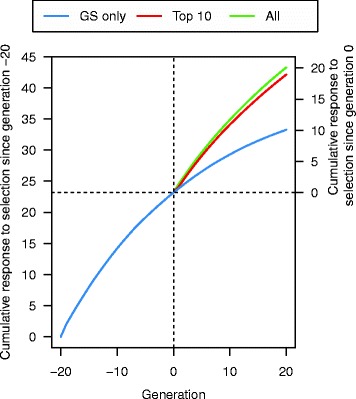
Cumulative response to selection across 21 generations of recent historical breeding based on genomic selection only (GS only) and 20 generations of future breeding based on GS only or genomic selection plus the promotion of alleles by genome editing (GS + PAGE) when 20 QTN were edited for all 25 sires or top 10 sires

GS + PAGE was effective for increasing response to selection for the quantitative trait. In comparison to GS only, editing all sires (A25se) for 20 QTNe per sire doubled the cumulative response to selection after both a few and many generations of selection (Fig. 3). For example, in generation 3, the cumulative response to selection was 2.09 units for GS only and 4.07 units for GS + PAGE, while in generation 20, the cumulative response to selection was 10.07 units for GS only and 20.09 units for GS + PAGE. However, this extra cumulative response to selection for GS + PAGE decreased as the number of QTNe per sire decreased (Table 1). For example, compared to the GS only scenario, the relative increase in cumulative response to selection after 20 generations in the GS + PAGE scenario was 2.00 and 1.08 times greater when editing A25se for 20 and one QTNe per sire, respectively. When using the same number of QTNe per sire, the scenarios for which all sires were edited (A25se) gave slightly greater cumulative response to selection compared to the scenarios for which only some sires were edited (T10se) (Fig. 4). Since there was no meaningful difference between the cumulative responses to selection of the T10se and B10se GS + PAGE scenarios, only the results for T10se are shown in Fig. 4.

**Table 1:** Cumulative response to selection (95 % confidence interval) relative to genomic selection only (GS only) across 20 generations of future breeding based on GS only or genomic selection plus the promotion of alleles by genome editing (GS + PAGE) when different numbers of sires and different numbers of QTN per sire were edited

Sires edited	Number of edits per sire				
	0 (GS only)	1 (1 QTNe)	5 (5 QTNe)	10 (10 QTNe)	20 (20 QTNe)
Bottom 10 (B10se)	1.00	1.04 (1.03–1.05)	1.23 (1.20–1.26)	1.46 (1.41–1.50)	1.89 (1.82–1.95)
Top 10 (T10se)		1.04 (1.03–1.05)	1.23 (1.20–1.26)	1.46 (1.41–1.50)	1.89 (1.82–1.95)
All 25 (A25se)		1.08 (1.06–1.09)	1.31 (1.28–1.35)	1.56 (1.51–1.61)	2.00 (1.93–2.08)

**Fig. 5:**
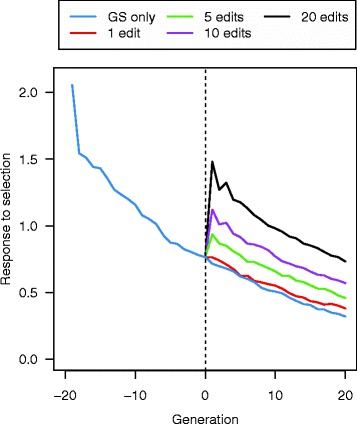
Response to selection between pairs of subsequent generations across 21 generations of recent historical breeding based on genomic selection only (GS only) and 20 generations of future breeding based on GS only or genomic selection plus the promotion of alleles by genome editing (GS + PAGE) when different numbers of QTN (1, 5, 10, or 20) were edited for all 25 sires

Quantifying response to selection from generation to generation revealed two trends (Fig. 5). First, the advantage of GS + PAGE over GS only was greater in the first few generations than in later generations because the segregating QTN with larger effects were used more effectively by GS + PAGE in the initial generations than by GS only. Second, in later generations, the relative contribution of PAGE to response to selection increased and the relative contribution to overall response to selection of GS decreased. This second trend arose because the contribution to response to selection declined more rapidly for GS than for PAGE, due to the greater impact of genetic drift and hitchhiking on the loss of favourable alleles in the GS only scenarios. For example, the response to selection for GS + PAGE for scenario A25se with 20 QTNe per sire was 1.91 times greater than response for GS only at generation 10 and 2.00 times greater at generation 20.

**Fig. 6:**
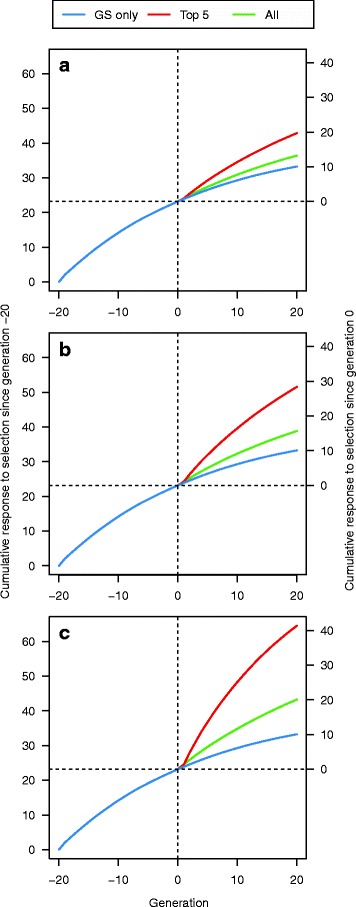
Cumulative response to selection across 21 generations of recent historical breeding based on genomic selection only (GS only) and 20 generations of future breeding based on GS only or genomic selection plus the promotion of alleles by genome editing (GS + PAGE) when **a** 125, **b** 250, or **c** 500 edits per generation were distributed across all of the 25 or the top 5 selected sires

When the total resources for PAGE were a limiting factor, the GS + PAGE scenarios in which a fixed number of QTNe per generation was distributed across a small number of sires, resulted in considerably more cumulative response to selection, compared to scenarios in which the same fixed number of QTNe per generation was distributed across a greater number of the sires (Fig. 6). For example, after 20 generations of GS + PAGE with 500 QTNe per generation, the cumulative response to selection for scenario T5se with 100 QTNe per sire was 4.12 times greater than that of GS only; while the cumulative response to selection for the scenario A25se with 20 QTNe per sire was only 2.00 times greater than that of GS only (Table 2). Therefore, the benefit of spreading the 500 QTNe per generation across the top five sires was 2.06 (i.e., 2.06 = 4.12/2.00) times greater than spreading them across all 25 sires. These trends were consistent across the range of total PAGE resources (i.e., totals of 125, 250, or 500 QTNe per generation). There was a noticeable difference in the shape of the response to selection across the generations of selection between scenarios A25se and the scenarios in which the PAGE resources were distributed across a small number of sires (T5se). In the first generation, distributing the edits across a small number of sires (T5se) never led to better results than distributing them across all sires (A25se) [See Additional file 1: Figure S1]. In the following generations, distributing the edits across a small number of sires improved response much more.

**Table 2:** Cumulative response to selection (95 % confidence interval) relative to genomic selection only (GS only) across 20 generations of future breeding based on GS only or genomic selection plus the promotion of alleles by genome editing (GS + PAGE) when the genome editing resources per generation were limited

Sires edited	Number of edits per generation			
	0 (GS only)	125 (25 QTNe)	250 (50 QTNe)	500 (100 QTNe)
Top 5 (T5se)	1.00	1.96 (1.89–2.04)	2.83 (2.71–2.96)	4.12 (3.95–4.29)
All 25 (A25se)		1.31 (1.28–1.35)	1.56 (1.51–1.61)	2.00 (1.93–2.08)

### Change in the frequency of favourable alleles

**Fig. 7:**
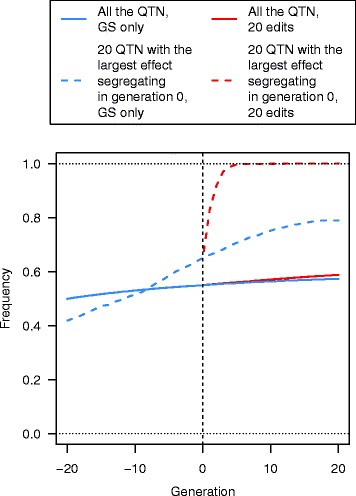
Mean frequency of the favourable alleles at all QTN that segregated at generation 0 or at the 20 QTN with the largest effect that segregated at generation 0 across 21 generations of recent historical breeding based on genomic selection only (GS only) and 20 generations of future breeding based on GS only or genomic selection plus the promotion of alleles by genome editing (GS + PAGE) when 20 QTN were edited in all of the 25 selected sires

In generation–20, the average frequency of favourable alleles at all QTN was 0.50. Selection using GS only increased the average frequency of favourable alleles by 0.05 from generation–20 to 0 (Fig. 7). Between generations 0 and 20, the frequency of favourable alleles increased by 0.02 for GS only and by 0.04 for the GS + PAGE scenario A25se with 20 QTNe per sire. GS only and GS + PAGE had different patterns of change in the average frequency of the favourable alleles at the QTN with the largest effects and these were also noticeably different from the change in the average frequency of the favourable alleles at all QTN. The average frequency of the favourable alleles at the 20 QTN with the largest effects for the GS + PAGE scenario A25se with 20 QTNe per sire, increased rapidly in the first three to four generations of GS + PAGE (Fig. 7). By generation 5, the average allele frequencies were already higher than 0.99, and full fixation was reached within 12 generations. For GS only, the favourable allele frequency of the 20 QTN with the largest effects increased approximately linearly up to generation 12, but at a considerably lower rate than for GS + PAGE scenarios. After generation 12, the rate of fixation of favourable alleles of the 20 QTN with the largest effects started to plateau at a value of less than 0.8 for GS only. As the number of QTNe increased, the trends of fixation for the other GS + PAGE scenarios became more similar to those for 20 QTNe per sire [See Additional file 2: Figure S2].

**Fig. 8:**
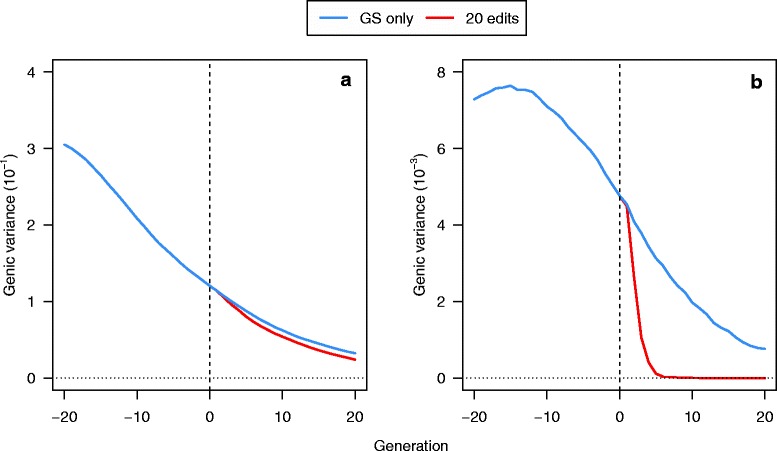
Genic variance due to (**a**) all the QTN that segregated at generation 0 or (**b**) the 20 QTN with the largest effect that segregated at generation 0 across 21 generations of recent historical breeding based on genomic selection only (GS only) and 20 generations of future breeding based on GS only or genomic selection plus the promotion of alleles by genome editing (GS + PAGE) when 20 edits were performed in all of the 25 selected sires

Changes in the frequencies of favourable alleles led to a reduction in the genic variance across generations. This drop was slightly larger for GS + PAGE than for GS only, as shown for GS only and GS + PAGE with A25se for 20 QTNe per sire in Fig. 8a. When focusing only on the genic variance due to the 20 QTN with the largest effects, a much larger drop was observed in the initial generations (i.e., generation 1 to 6) for GS + PAGE than for GS only (Fig. 8b). The genic variance due to the 20 QTN with the largest effects increased from generation–20 to generation–15 because these large QTN that were very rare in the base generation 0 and GS only selection caused an increase in their frequency in the initial generations.

### Number of distinct QTN edited and their genic variance and effect sizes

**Fig. 9:**
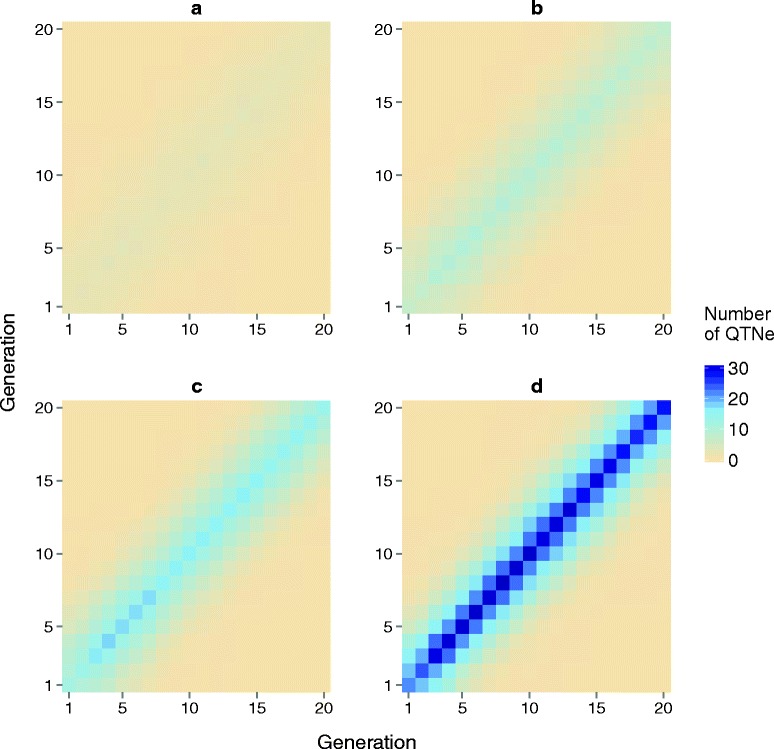
Number of distinct QTN being edited (diagonal) and the number of distinct QTN being edited that were in common between pairs of generations (off-diagonal) across the 20 future generations of breeding based on the genomic selection plus the promotion of alleles by genome editing (GS + PAGE) when **a** 1, **b** 5, **c** 10, or **d** 20 edits were performed in all of the 25 selected sires

The number of distinct QTNe in each generation and the number of distinct QTNe that were shared between pairs of generations for A25se are in Fig. 9. The number of distinct QTNe increased up to generation 3 and then remained stable. As the number of QTNe per sire increased from 1 (Fig. 9a) to 20 (Fig. 9d), so did the number of distinct QTNe per generation. For pairs of generations that were more distant from each other, the number of distinct QTNe that were common decreased.

The number of distinct QTNe across all 20 generations of GS + PAGE increased with the number of QTNe per sire and with the number of sires being edited (Table 3). For example, for scenario A25se, the average number of distinct QTNe across all 20 generations was 18.1 for one QTNe per sire and 314.6 for 20 QTNe per sire. For scenarios with 20 QTNe per sire, the average number of distinct QTNe across the 20 generations was 284.3 when only 10 sires (either T10se or B10se) were edited and 314.6 when all 25 sires were edited (A25se). When the total editing resources were limited, the number of distinct QTNe across the 20 generations of GS + PAGE was much greater when these resources were focussed on a small number of sires as opposed to all sires (Table 4). For example, for scenarios with 500 QTNe in a given generation, a total of 1251.7 distinct QTNe were observed across the 20 generations for scenario T5se but only 314.6 distinct QTNe for scenario A25se.

**Table 3:** Number of distinct QTN that were edited (95 % confidence interval) across 20 future generations of breeding based on genomic selection plus the promotion of alleles by genome editing (GS + PAGE) when different numbers of sires and different numbers of QTN per sire were edited

Sires edited	Number of edits per sire			
	1 (1 QTNe)	5 (5 QTNe)	10 (10 QTNe)	20 (20 QTNe)
Bottom 10 (B10se)	14.0 (12.0–14.0)	69.1 (66.0–72.2)	142.3 (138.0–146.6)	284.3 (277.4–291.2)
Top 10 (T10se)	14.0 (12.0–14.0)	69.1 (66.0–72.2)	142.3 (138.0–146.6)	284.3 (277.4–291.2)
All 25 (A25se)	18.1 (15.4–20.8)	82.6 (79.4–85.8)	157.9 (151.3–164.5)	314.6 (304.9–324.3)

**Table 4:** Number of distinct QTN that were edited (95 % confidence interval) across 20 generations of future breeding based on genomic selection plus the promotion of alleles by genome editing (GS + PAGE) when the genome editing resources per generation were limited

Sires edited	Number of edits per generation		
	125 (25 QTNe)	250 (50 QTNe)	500 (100 QTNe)
Top 5 (T5se)	312.6 (306.6–318.6)	627.9 (617.6–638.2)	1251.7 (1238.4–1265.0)
All 25 (A25se)	82.6 (79.4–85.8)	157.9 (151.3–164.5)	314.6 (304.9–324.3)

When generation 0 was assumed to be the base population, the amount of genic variance that the QTNe explained in a given scenario closely matched the number of QTNe in that scenario (Table 5). As the number of QTNe per sire increased, the genic variance explained by the QTNe increased. In addition, when the total editing resources were limited, the genic variance explained by the QTNe in a given scenario matched closely the number of distinct QTNe in that scenario (Table 6). For example, for scenarios with 500 QTNe in a given generation, the genic variance of the QTNe across the 20 generations was 71.0 % of the total genic variance in generation 0 for scenario T5se but only 36.0 % for scenario A25se.

**Table 5:** Proportion of genic variance in generation 0 explained (95 % confidence interval) by the distinct QTN that were edited across 20 generations of future breeding based on genomic selection plus the promotion of alleles by genome editing (GS + PAGE) when different numbers of sires and different numbers of QTN per sire were edited

Sires edited	Number of edits per sire			
	1 (1 QTNe)	5 (5 QTNe)	10 (10 QTNe)	20 (20 QTNe)
Bottom 10 (B10se)	3.4 (2.7–4.2)	12.4 (11.1–13.6)	20.8 (19.2–22.4)	33.6 (31.4–35.8)
Top 10 (T10se)	3.4 (2.7–4.2)	12.4 (11.1–13.6)	20.8 (19.2–22.4)	33.6 (31.4–35.8)
All 25 (A25se)	4.3 (3.4–5.3)	14.3 (12.9–15.7)	22.5 (20.5–24.5)	36.0 (33.8–38.2)

**Table 6:** Proportion of genic variance in generation 0 explained (95 % confidence interval) by the distinct QTN that were edited across 20 generations of future breeding based on genomic selection plus the promotion of alleles by genome editing (GS + PAGE) when the genome editing resources per generation were limited

Sires edited	Number of edits per generation		
	125 (25 QTNe)	250 (50 QTNe)	500 (100 QTNe)
Top 5 (T5se)	34.8 (32.6–37.0)	52.0 (49.8–54.3)	71.0 (69.3–72.7)
All 25 (A25se)	14.3 (12.9–15.7)	22.5 (20.5–24.5)	36.0 (33.8–38.2)

**Fig. 10:**
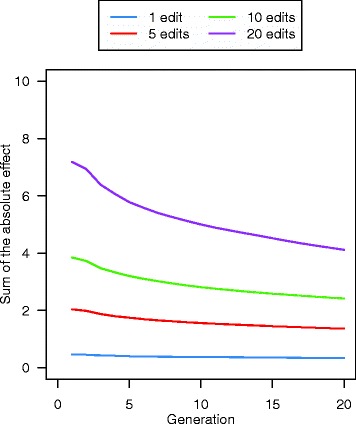
The sum of the absolute effects of the edited QTN across the 20 future generations of breeding based on the genomic selection plus the promotion of alleles by genome editing (GS + PAGE) when 1, 5, 10, or 20 QTN were edited in all of the 25 selected sires

The QTN that were edited in the initial generations of GS + PAGE had larger effect sizes than those that were edited in later generations, which resulted in a greater response to selection in earlier generations with PAGE than in later generations. For example, in the scenario with 20 QTNe per sire, the reduction in the sum of the absolute effects of the QTN that were edited was large in the first few generations but became smaller thereafter and then stabilised (Fig. 10).

The number of times a particular QTN was edited was strongly related to the frequency of the favourable allele in the population. When the frequency of the favourable allele at a QTN with a large effect was small in the base population, it was edited often. For example, out of the 40 analyses (i.e., 10 replicates for four scenarios (1, 5, 10, and 20 QTNe per sire) with A25se), the frequency of the favourable allele in the base population at the QTN with the largest effects was less than 0.10 in a total of eight replicates and greater than 0.90 also in another eight replicates. For the former eight replicates, all 25 sires were edited for this QTN, while a maximum of three sires were edited for this QTN for the latter eight replicates.

### Inbreeding coefficients

**Fig. 11:**
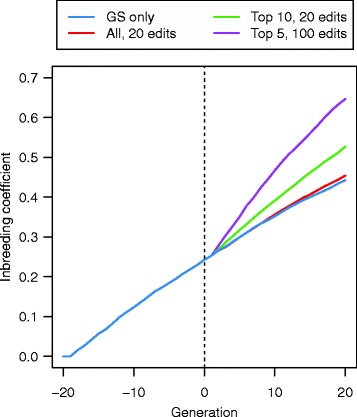
Inbreeding coefficient across 21 generations of recent historical breeding based on genomic selection only (GS only) and 20 generations of future breeding based on GS only or genomic selection plus the promotion of alleles by genome editing (GS + PAGE) when 20 edits were performed in all of 25 or the top 10 sires or 100 edits were performed on the top five sires

The average coefficient of inbreeding increased with each generation (Fig. 11). There was almost no difference in the rate of inbreeding between GS only and the GS + PAGE scenarios in which all sires were edited (i.e., A25se). However, GS + PAGE scenarios in which only a subset of the sires were edited resulted in a large increase in the rate of inbreeding compared to when all sires were edited. In addition, the rate of inbreeding for scenarios in which only five sires were edited (T5se) was much greater than for scenarios in which 10 sires were edited (T10se). After 20 generations of selection, the average inbreeding coefficient in generation 20 was 0.44 for GS only, 0.45 for GS + PAGE scenario A25se with 20 QTNe per sire, 0.53 for GS + PAGE scenario T10se with 20 QTNe per sire, and 0.65 for GS + PAGE scenario T5se with 100 QTNe per sire. There was no difference in inbreeding level between scenarios for which the top 10 (T10se) or bottom 10 (B10se) sires were edited (results not shown).

## Discussion

The results of this study show that PAGE has great potential as a tool for increasing response to selection of quantitative traits in livestock breeding programs. Compared to GS only, GS + PAGE resulted in large increases in response to selection. These increases were observed both in the short-and long-term. When editing resources were limited, performing more edits on each of a smaller number of sires resulted in greater response to selection but also much greater increases in inbreeding than performing fewer edits on each of a larger number of sires. When focussing the editing resources on this smaller number of sires, there was no difference in response to selection or inbreeding whether the edited sires were those with the highest or lowest breeding values amongst the selected set.

When considering all QTN, almost no differences were observed in the rate of change in allele frequencies between GS only and GS + PAGE. However, when only the 20 QTN with the largest effects were considered, there were large differences in changes in allele frequencies. With GS only, the average frequency of favourable alleles at these 20 QTN plateaued at less than 0.8 within 20 generations of selection because some of the favourable alleles were lost due to drift or hitchhiking before they could be driven to fixation by selection. This loss of favourable alleles with large effects did not occur with GS + PAGE. Changes in the frequency of favourable alleles led to a drop in the genic variance across generations, which was larger for GS + PAGE than for GS only.

The total number of distinct QTNe in a given generation and cumulatively across generations was affected by the editing strategy. The cumulative total number of distinct QTNe across the 20 generations was as low as 14 when only a small number of QTNe were performed per generation and these edits were focussed on only a few sires, and was as high as 1252 when a large number of QTNe were performed per generation and on a small number of sires. The genic variance that was explained by the distinct QTNe across the 20 generations of future breeding, increased with the number of QTNe per sire but varied greatly between scenarios, from 1 % to 71 % of the genic variance present in generation 0. The relative benefit of PAGE over GS only in terms of response to selection increased over generations and the absolute sum of the effects of the QTNe in a given generation decreased over generations. The number of QTNe per sire affected both of these trends. When the editing resources were distributed equally across all sires, the coefficient of inbreeding was basically the same for GS + PAGE and GS only, but when only a portion of sires was edited, the coefficient of inbreeding increased.

Compared to GS only, GS + PAGE with relatively small numbers of QTNe (e.g., 20 QTNe per sire) was effective for increasing response to selection for a quantitative trait defined by thousands of QTN, each with relatively small effects. It appears that the primary driver of the benefit of GS + PAGE over GS only was the rapid change in frequencies of favourable alleles at the QTN with the larger of these small effects; which both increased their rate of fixation and prevented favourable alleles at these QTN from being lost from the population due to drift and hitchhiking. Even for a quantitative trait that is influenced by thousands of QTN, with small effects sampled from a normal distribution, there is a small number of QTN that have noticeably larger effects. It is these QTN that PAGE uses primarily. GS only, even with perfect accuracy, cannot shift the frequencies of these alleles sufficiently to be as effective as GS + PAGE. The ability of GS only, even with perfect accuracy, to fix favourable alleles quickly is limited by the small levels of recombination that naturally occur in livestock populations. In the presence of many QTN, low levels of recombination limit the opportunity of all of the favourable alleles to occur together in the selected individuals. If selection with GS only focussed on attempting to combine even a small number of the QTN with the largest effects together, much of the response to selection would be lost due to the reduced selection pressure on the rest of the QTN. In this context, PAGE can be conceptualised as targeted and controlled inflation of recombination that helps to combine these large QTN, while maintaining selection pressure on all other QTN.

The increase in inbreeding was much greater when a fixed amount of PAGE resources was focused on a small subset of sires than when the PAGE resources were spread evenly across all sires, which resulted in basically the same level of inbreeding as that obtained with GS only. This greater inbreeding was the same for scenarios for which the subset of sires that were edited included the top or bottom sires (T10se or B10se) and was caused by the very large contributions that the progeny of the subset of edited sires make to future generations. Although all 25 sires had equal contributions to the next generation, the progeny of the edited sires had significantly better genetic merit than the progeny of non-edited sires and, therefore, formed a much greater proportion of the individuals from which the next-generation sires were selected, which contributed to the greater inbreeding when only a portion of sires were edited. Effectively the edited sires became super-grandsires.

This study used simulated data that made a number of assumptions: (i) the trait was entirely defined by additive QTN, (ii) the QTN and their effects were known without error, (iii) GS had perfect accuracy, and (iv) genome editing had perfect precision and there was no off-target editing. While some of these assumptions are unrealistic, we believe that they do not invalidate the overall conclusion of the study, i.e. that GS + PAGE is a powerful method to increase response to selection for quantitative traits. Currently, perhaps the most debatable assumption is that all QTN can be discovered with perfect precision, which is not possible with livestock datasets of the size that are presently available. However, our results show that only the QTN with the largest effects need to be discovered. Finding such QTN may be possible in very large datasets and several initiatives are underway to generate massive datasets in livestock breeding programs with the explicit aim of having datasets of sufficient size and sequence information to enable large numbers of QTN to be discovered, e.g. [31]. A recent study on 253 000 human individuals who were genotyped at high-density showed that GWAS was able to find 697 “hits” that together explained 20 % of the heritability of human height [26] and smaller studies in other traits show similar trends [32, 33]. While some GWAS hits have been functionally validated and replicated across distinct populations, functional validation on a wide scale remains a major challenge. In our study, the number of distinct QTNe was less than 35 in any given generation and less than 320 across all 20 generations when using 20 or less QTNe per sire. This suggests that it may be feasible to detect a sufficient number of QTN to enable PAGE to be used to generate the type of benefit that was observed in this study. Furthermore, human GWAS datasets are often designed to detect common QTN that tend to have moderate effect sizes. Large livestock datasets that have large half-sib and cousin family structures can detect common QTN with smaller effect sizes, and are also likely more effective in identifying rare QTN with large effects (including relatively recent mutations). Rare mutations with large effects could be a valuable source of targets for PAGE.

Major improvements have been made in the precision of GE during the last few years. Targeting specific nucleotides was significantly improved with ZFN and TALEN and the whole process was simplified with the latest development in the technology based on CRISPR. The number of off-target effects has been considerably reduced [34]. The main challenge for the future will be to improve the rate of success of the production of live edited animals, since, currently, the percentage of successfully edited animals is low. In spite of these challenges and some regulatory unknowns, we anticipate that PAGE will be a widely used and accepted technology within the foreseeable future.

In this work, a relatively simple strategy was used to prioritise the alleles that were to be edited, i.e., alleles with the largest effects in the sires. Applying PAGE in a selected set of sires seemed a prudent approach because of the current high costs of PAGE but there may be more optimal strategies. For example, the alleles could be prioritised based on their contribution to the total genic variance, by prioritising on the average allele substitution effect, which is influenced by the effect size and allele frequency. The allele frequency used to prioritise QTN for editing could be the frequency in the base population or the current generation. Using the current allele frequencies would emphasize favourable alleles with large effects that are rare at the time of editing and which could be edited to maximise the short-term response. In addition, the location of QTN along the genome relative to each other and to the distribution of recombinations, *i.e.*, recombination hotspots, could be accounted for when prioritising alleles for editing. Finally, the impact of PAGE could be optimised for each generation by performing in-silico PAGE, i.e. testing many different permutations of the distribution of edits across the population, in order to identify a set of edits that would maximise the benefits.

Prioritizing QTN for PAGE based on allele frequencies raises an interesting point in terms of editing standing or historical variation within a population or even variation from other populations. In this study, PAGE was developed to explicitly focus on the promotion of favourable alleles that are already present in a population but there are other sources of favourable alleles. Favourable alleles may have been lost from a population, may have never existed in a population but be present in different populations of the same species, may exist in other species, or could be synthesised. As biological knowledge increases and the use of genome editing becomes more efficient, it may be interesting to explore sources of favourable alleles that are beyond the population under selection.

Further work is also needed to optimise PAGE strategies in terms of which individuals should be edited, as opposed to choosing which of the QTN should be edited in a sire. While generating ‘super grandsires’ has a major benefit for driving response to selection, it also causes a major increase in the rate of inbreeding. Thus, in order to make PAGE a tool that can be used in breeding programs that are sustainable in the long-term, it will be necessary to develop breeding programs that enable this level of response to selection to be achieved while reducing the corresponding increases in inbreeding.

## Conclusions

PAGE is a new technique with a potential future application for increasing responses to selection for quantitative traits in livestock breeding programs. Simulations used in this study showed that the application of GS + PAGE could achieve substantial improvements in response to selection over GS only. The power of PAGE derives from the fact that it enables favourable alleles at QTN to be selected for independently of the haplotypes, chromosomes, and individuals that carry them; this means that the barrier of the limited number of recombinations that occur during meiosis can be overcome. Successful use of PAGE in breeding programs requires the discovery of the QTN that underlie quantitative traits and this, in turn, requires larger datasets than what is currently available. It will also be necessary to develop breeding programs that optimally implement PAGE, to ensure that PAGE does not result in a rapid depletion of the genetic variance in a population.

## Additional files

Additional file 1: Figure S1. Response to selection between pairs of subsequent generations across 21 generations of recent historical breeding based on genomic selection only (GS only) and 20 generations of future breeding based on GS only or genomic selection plus the promotion of alleles by genome editing (GS + PAGE) when (a) 125, (b) 250, and (c) 500 QTN were edited per generation for the top 5 or all of the 25 selected sires. Additional file 2: Figure S2. Allele frequency of the 20 QTN with the largest effect that still segregated in generation 0 across 21 generations of recent historical breeding based on genomic selection only (GS only) and 20 generations of future breeding based on GS only or genomic selection plus the promotion of alleles by genome editing (GS + PAGE) when 1, 5, or 10 QTN were edited for all 25 selected sires.
