# Erratum to: Potential of promotion of alleles by genome editing to improve quantitative traits in livestock breeding programs

**DOI:** 10.1186/s12711-015-0144-2

**Published:** 2015-09-11

**Authors:** Janez Jenko, Gregor Gorjanc, Matthew A. Cleveland, Rajeev K. Varshney, C. Bruce A. Whitelaw, John A. Woolliams, John M. Hickey

**Affiliations:** The Roslin Institute and Royal (Dick) School of Veterinary Studies, The University of Edinburgh, Easter Bush, Midlothian, Scotland UK; Genus plc., 100 Bluegrass Commons Blvd., Suite 2200, Hendersonville, TN 37075 USA; International Crop Research Institute for the Semi-Arid Tropics (ICRISAT), Patancheru, India

After the publication of this work [1], we noticed that Figs. 1 and 2 were accidentally interchanged. The correct version order of Figs. 1 and 2 are provided here. The original article was corrected.

**Fig. 1:**
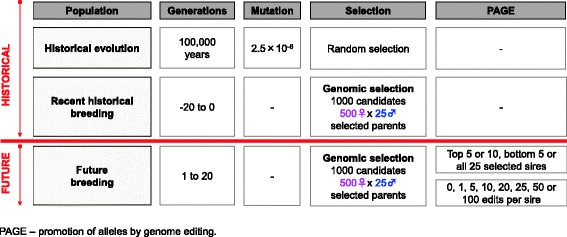
Simulated scenarios for promotion of alleles by genome editing

**Fig. 2:**
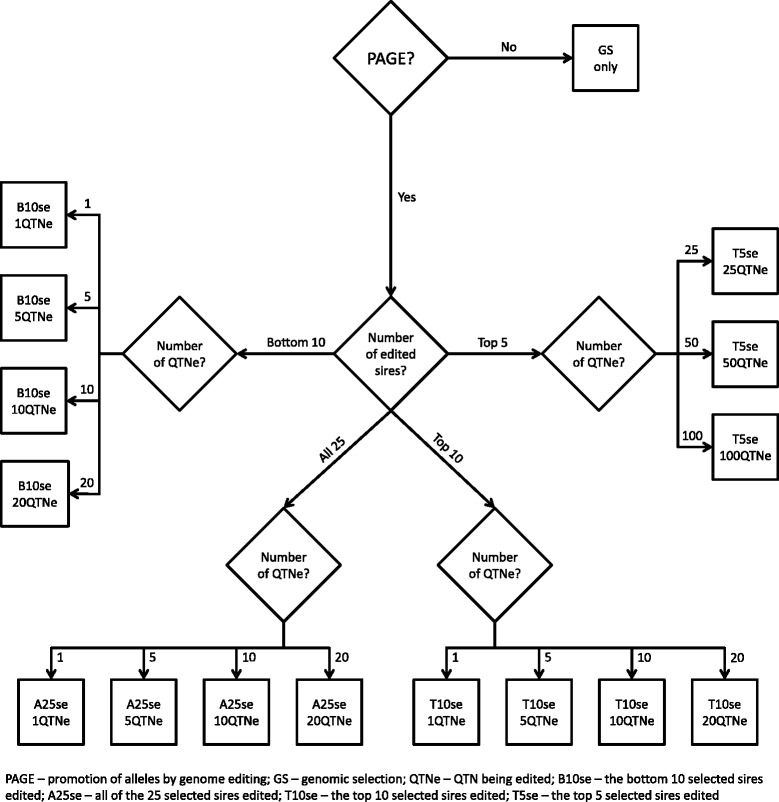
Overall design of the simulation
