# Supplementary material for: Potential of promotion of alleles by genome editing to improve quantitative traits in livestock breeding programs
Source: Genet Sel Evol. 2015 Jul 2;47(1):55. doi: 10.1186/s12711-015-0135-3 (PMC4487592; doi:10.1186/s12711-015-0135-3)
Supplement: Additional file 1: Figure S1. — Response to selection between pairs of subsequent generations across 21 generations of recent historical breeding based on genomic selection only (GS only) and 20 generations of future breeding based on GS only or genomic selection plus the promotion of alleles by genome editing (GS + PAGE) when (a) 125, (b) 250, and (c) 500 QTN were edited per generation for the top 5 or all of the 25 selected sires. [file 12711_2015_135_MOESM1_ESM.pdf]

Additional file 1 Figure S1

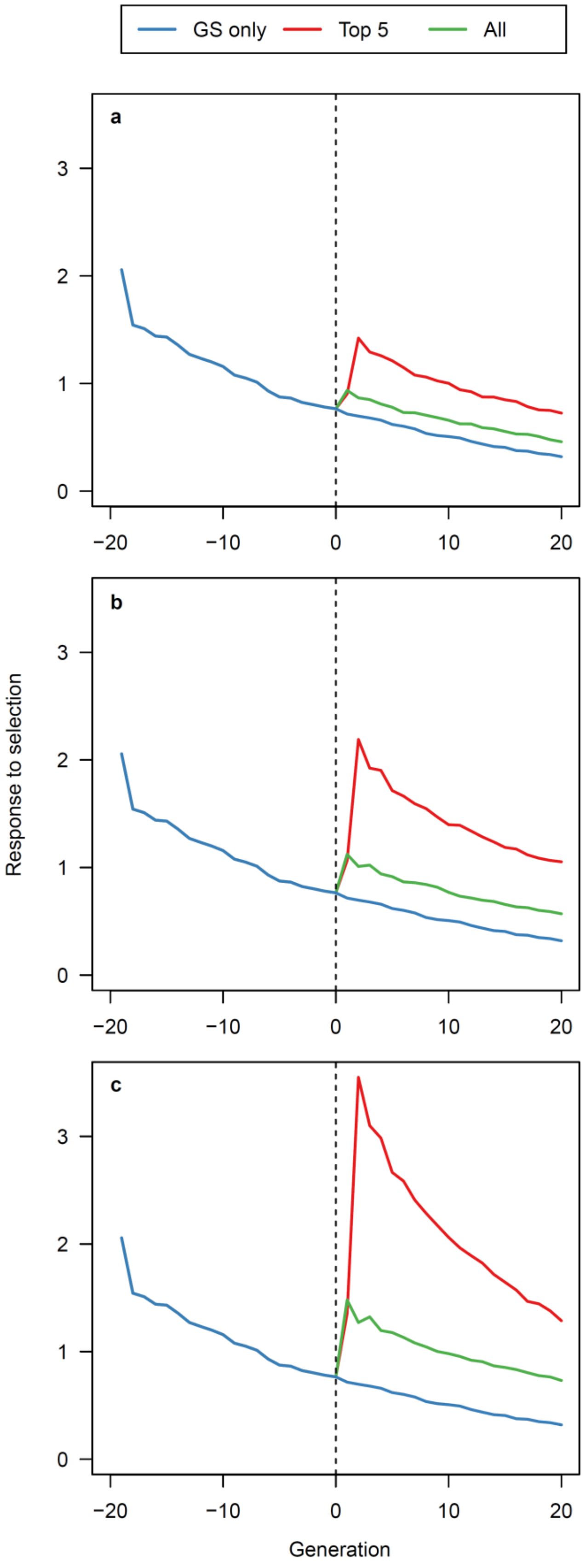

Title: Response to selection between pairs of subsequent generations across 21 generations of recent historical breeding based on genomic selection only (GS only) and 20 generations of future breeding based on GS only or genomic selection plus the promotion of alleles by genome editing (GS+PAGE) when (a) 125, (b) 250, and (c) 500 QTN were edited per generation for the top 5 or all of the 25 selected sires.
