# Supplementary material for: Potential of promotion of alleles by genome editing to improve quantitative traits in livestock breeding programs
Source: Genet Sel Evol. 2015 Jul 2;47(1):55. doi: 10.1186/s12711-015-0135-3 (PMC4487592; doi:10.1186/s12711-015-0135-3)
Supplement: Additional file 2: Figure S2. — Allele frequency of the 20 QTN with the largest effect that still segregated in generation 0 across 21 generations of recent historical breeding based on genomic selection only (GS only) and 20 generations of future breeding based on GS only or genomic selection plus the promotion of alleles by genome editing (GS + PAGE) when 1, 5, or 10 QTN were edited for all 25 selected sires. [file 12711_2015_135_MOESM2_ESM.pdf]

Additional file 2 Figure S2

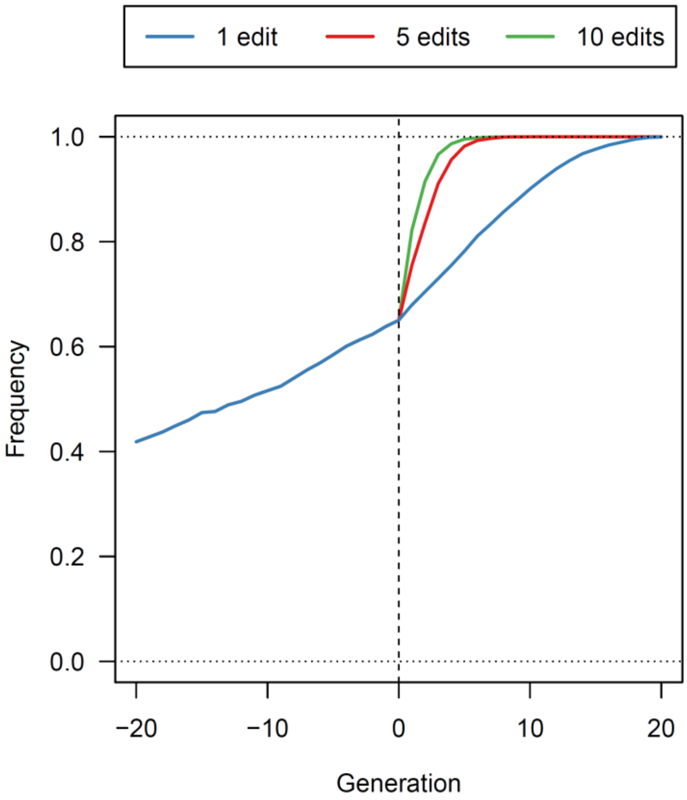

Title: Allele frequency of the 20 QTN with the largest effect that still segregated in generation 0 across 21 generations of recent historical breeding based on genomic selection only (GS only) and 20 generations of future breeding based on GS only or genomic selection plus the promotion of alleles by genome editing (GS+PAGE) when 1, 5, or 10 QTN were edited for all 25 selected sires.
